# The probability of patients being admitted from the emergency department is negatively correlated to in-hospital bed occupancy – a registry study

**DOI:** 10.1186/1865-1380-7-8

**Published:** 2014-02-05

**Authors:** Mathias C Blom, Fredrik Jonsson, Mona Landin-Olsson, Kjell Ivarsson

**Affiliations:** 1 IKVL, Lund University, IKVL/Sektion I-II, Akutmedicin, Hs 32, EA-blocket, plan 2, Universitetssjukhuset, 221 85 Lund, Sweden; 2 Pre och intrahospital akutsjukvård, Helsingborgs lasarett, S Vallgatan 5, Helsingborg 251 87, Sweden

**Keywords:** Bed occupancy, Emergency care, Patient admission

## Abstract

**Background:**

The association between emergency department (ED) overcrowding and poor patient outcomes is well described, with recent work suggesting that the phenomenon causes delays in time-sensitive interventions, such as resuscitation. Even though most researchers agree on the fact that admitted patients boarding in the ED is a major contributing factor to ED overcrowding, little work explicitly addresses whether in-hospital occupancy is associated to the probability of patients being admitted from the ED. The objective of the present study is to investigate whether such an association exists.

**Methods:**

Retrospective analysis of data on all ED visits to Helsingborg General Hospital in southern Sweden between January 1, 2011, and December 31, 2012, was undertaken. The fraction of admitted patients was calculated separately for strata of in-hospital occupancy <95%, 95–100%, 100–105%, and >105%. Multivariate models were constructed in an attempt to take confounding factors, e.g., presenting complaints, age, referral status, triage priority, and sex into account. Subgroup analysis was performed for each specialty unit within the ED.

**Results:**

Overall, 118,668 visits were included. The total admitted fraction was 30.9%. For levels of in-hospital occupancy <95%, 95–100%, 100–105%, and >105% the admitted fractions were 31.5%, 30.9%, 29.9%, and 28.7%, respectively. After taking confounding factors into account, the odds ratio for admission were 0.88 (CI 0.84–0.93, *P* >0.001) for occupancy level 95–100%, 0.82 (CI 0.78–0.87, *P* >0.001) for occupancy level 100–105%, and 0.74 (CI 0.67–0.81, *P* >0.001) for occupancy level >105%, relative to the odds ratio for admission at occupancy level <95%. A similar pattern was observed upon subgroup analysis.

**Conclusions:**

In-hospital occupancy was significantly associated with a decreased odds ratio for admission in the study population. One interpretation is that patients who would benefit from inpatient care instead received suboptimal care in outpatient settings at times of high in-hospital occupancy. A second interpretation is that physicians admit patients who could be managed safely in the outpatient setting, in times of good in-hospital bed availability. Physicians thereby expose patients to healthcare-associated infections and other hazards, in addition to consuming resources better needed by others.

## Background

The association between emergency department (ED) overcrowding and poor patient outcomes is well described [1-7]. Recent work suggests that ED overcrowding compromises timeliness of resuscitative care, with potentially devastating effects to individual patients [8], and that ED overcrowding might have increased in magnitude over time [9]. Strategies to reduce overcrowding have been proposed [2,10-15] but their extent of implementation is variable [4,14,16].

Boarding of admitted patients in the ED has been highlighted several times as the major cause of ED overcrowding [1,2,14,17-19]. Boarding is, in turn, caused by scarcity of inpatient beds [1,2,14,16,17]. Some consider ED overcrowding a symptom of the broader dysfunction in the healthcare system, rather than a problem residing solely in the ED [20-22]. Although not thoroughly investigated, full capacity protocols and other solutions aiming at distributing workload throughout the hospital instead of accumulating patients in the ED appear promising [16,23]. When asked, patients prefer boarding in inpatient hallways to ED hallways [24]. Other specific solutions to reduce boarding include synchronizing discharges from inpatient wards with admission peaks [25] and to eliminate bottlenecks delaying discharge, e.g., availability of surgery [26].

Admission is a comparably expensive intervention for ED patients, the use of which needs to be scrutinized in order to better understand cost-effectiveness in the evolving role of the EDs in Sweden and worldwide [21]. There is some evidence for physicians avoiding hospital admissions as an adaptive strategy in crowded conditions [21,27], but few studies have explicitly addressed the correlation between in-hospital occupancy and the probability of admission. Beds are often scarce in Swedish hospitals, inciting the Swedish National Board of Health and Welfare to make the matter subject to national follow-up since 2012.

The objective of the present study is to test whether the probability of being admitted from the ED is correlated to the in-hospital occupancy at the time of patient presentation in the ED. The study is primarily hypothesis-generating and constitutes part of an extensive project designed to elucidate the effects of high in-hospital occupancy on the treatment of acutely ill patients in the country council region of Skåne in southern Sweden.

## Methods

### Study design

This registry study included all visits to the ED of Helsingborg General Hospital registered in the ED information system Patientliggaren® between 1^st^ January 2011 and 31^st^ December 2012, not resulting in referral to another hospital. No further selection was made, in order not to introduce selection bias.

### Setting

Helsingborg General Hospital is one of four emergency hospitals in the region of Skåne in southern Sweden. It is a 420-bed hospital with an ED serving a population of around 250,000. Due to tourism, the population expands to nearly 300,000 during the summer. The annual ED census is around 60,000, with approximately 15% of patients arriving by ambulance. Patients are registered in the information system Patientliggaren® by a secretary upon arrival. Patients who arrive by ambulance or are referred by a physician gain access to the ED after registration. Non-referred patients are given access to the ED after registration in accordance with pre-defined guidelines or are further evaluated by a nurse in primary triage. Such cases could be referred elsewhere (e.g., to primary care). About 10–15% of all visits enter the ED via primary triage. After entering the ED, secondary triage is performed by a nurse. The 4-level triage system “medical emergency triage and treatment system” was used during the study period. It addresses the medical urgency of a case by evaluating the main complaint and vital parameters. The triage priority number is registered in Patientliggaren® directly after secondary triage. Only physicians may down-prioritize patients.

Patients are directed to separate units for Surgery, Orthopaedics, Medicine, and Otolaryngology in a triage-to-specialty model after secondary triage. A complementary unit staffed by emergency physicians capable of handling all complaints except for psychiatric, otolaryngologic, ophthalmologic, and paediatric (medicine) was also introduced in 2010 and operates from 8 am to 11 pm daily. There are separate EDs for children with medical conditions (<18 years of age) and for patients with obstetric/gynaecologic, psychiatric or ophthalmologic complaints. These are not included in the study. Patients with suspected hip-fractures and some geriatric patients with high acuity conditions are admitted directly and bypass the ED. Hand surgery, neurosurgery, and thoracic surgery is not available at the hospital. The availability of endovascular surgery and percutaneous coronary intervention is limited afterhours (between 17.00–08.00) and during weekends. Patients with these needs are referred to Skane University Hospital in Lund. Swedish national reimbursement systems are tied to a goal of 80% of visits with ED length of stay ≤4 hours. ED length of stay at Helsingborg hospital is shorter than in academic EDs overseas [4,28]. Access to emergency care in Sweden is not dependent on private insurance status.

### Data sources

Data on referral status was retrieved from the in-hospital information system PASiS. Data on all other variables was retrieved from the ED information system Patientliggaren®. Matching was performed by the hospital informatics unit using QlikView® software. Data on in-hospital bed occupancy was retrieved from the informatics unit and was matched by the author (MB) using IBM® SPSS® Statistics 19. Data in Patientliggaren® has been validated by the regional epidemiological unit “EpiCentrum” of the region of Skåne, as part of another project, in 2012–2013.

### Statistical analysis

The admitted fraction was computed for strata of in-hospital occupancy of <95%, 95–100%, 100–105%, and >105%. Subgroup analysis was performed for each specialty unit.

Binary logistic regression models were developed in an attempt to take confounding factors into account. Clinical judgement governed the decision of which predictors to screen for inclusion, but was inevitably tainted by data availability. Decisions were made *a priori* to analysis. Screened variables were the 10 most common presenting complaints, age-group, referral status, triage priority, presentation on a shift experiencing many visits (see definition below), presentation on night shift and during weekends, sex, leaving without being seen (LWBS), entering ED via primary triage, time to physician and in-hospital occupancy.

Age was grouped into intervals 0–18 years, 18–40 years, 40–64 years, and ≥65 years; youths in Sweden become of age at 18 and pension-age is 65 years. The youngest two age categories were merged in the analysis of the medicine unit, as children with medical conditions are assessed in a separate ED. In-hospital occupancy was categorized as <95%, 95–100%, 100–105%, and >105%. Presentation on a shift experiencing many visits was constructed as a dichotomous variable indicating presentation on one of the 25% of shifts subject to most visits (adjusted for shift type and unit). The night shift was set from 00.00 am to 08.00 am. Reference intervals for categorical predictors were in-hospital occupancy <95%, triage priority 3, age ≥65 years, presenting complaint other than the 10 most common, and not being referred to the ED. Since causes of missing data were not known, imputation was not considered an option and missing data was instead indicated by a separate category in each model [29].

Predictors were tested for crude association with the outcome before entering the preliminary primary effects model. Associations weaker than *P* = 0.25, but of clinical importance, were still included [30]. Multicollinearity testing was performed using Spearman correlation [31]. Selection of interaction terms screened for inclusion in the final models was governed by clinical significance and terms were added to models one by one rather than stepwise [30]. Model fit was evaluated through Nagelkerke’s R^2^. The association between each predictor and the outcome were addressed by the -2LL and the Wald statistics. Models were screened for influential cases by addressing standardized residuals and Cook’s distance.

Data was anonymized before entering SPSS. Ethical approval was granted by the ethics committee of Lund in February 6, dnr 2013/11.

## Results

Between January 1, 2011 and December 31, 2012, 120,203 visits to the medicine, surgery, orthopaedics, emergency medicine, and otolaryngology units were registered in Patientliggaren®; 118,668 visits did not result in referral to another hospital and were included in the study. Of these, 35,016 were directed to the medicine unit, 27,710 to the surgery unit, 27,600 to the orthopaedics unit, 16,740 to the emergency physician unit, and 11,602 to the otolaryngology unit. The admitted fraction was 30.9% overall, 47.2% for the medicine unit, 32.8% for the surgery unit, 14.9% for the orthopaedics unit, 30.8% for the emergency physician unit, and 14.8% for the otolaryngology unit. Detailed descriptive data are reproduced in Table 1.

**Table 1:** Descriptive data

		**All units**	**Medicine unit**	**Surgery unit**	**Orthopaedics unit**	**Emergency physician unit**	**Otolaryngology unit**
**Admission status**	Not admitted	82,051 (69.1%)	18,493 (52.8%)	18,608 (67.2%)	23,484 (85.1%)	11,580 (69.2%)	9,886 (85.2%)
	Admitted	36,617 (30.9%)	16,523 (47.2%)	9,102 (32.8%)	4,116 (14.9%)	5,160 (30.8%)	1,716 (14.8%)
**Age (yrs)**	0–17	16,381 (13.8%)	7,914 (22.6%)	4,846 (17.5%)	6,231 (22.6%)	1,747 (10.4%)	3,508 (30.2%)
	18–39	30,097 (25.4%)		7,684 (27.7%)	7,442 (27.0%)	4,087 (24.4%)	3,019 (26.0%)
	40–64	33,468 (28.2%)	11,139 (31.8%)	7,328 (26.4%)	7,372 (26.7%)	4,855 (29.0%)	2,774 (23.9%)
	>65	38,722 (32.6%)	15963 (45.6%)	7,852 (28.3%)	6,555 (23.8%)	6,051 (36.1%)	2,301 (19.8%)
**Referral status**	Not referred	91,168 (76.8%)	27,205 (77.7%)	22,187 (80.1%)	21,461 (77.8%)	12,698 (75.9%)	7,617 (65.7%)
	Referred	18,667 (15.7%)	5,052 (14.4%)	3,379 (12.2%)	4,265 (15.5%)	3,156 (18.9%)	2,815 (24.3%)
	Missing	8,833 (7.4%)	2,759 (7.9%)	2,144 (7.7%)	1,874 (6.8%)	886 (5.3%)	1,170 (10.1%)
**Triage priority**	1	5,689 (4.8%)	3,298 (9.4%)	1,437 (5.2%)	277 (1.0%)	587 (3.5%)	90 (0.8%)
	2	18,461 (15.6%)	9,136 (26.1%)	3,898 (14.1%)	2,181 (7.9%)	2,626 (15.7%)	620 (5.3%)
	3	63,828 (53.8%)	17,423 (49.8%)	16,730 (60.4%)	15,522 (56.2%)	9,670 (57.8%)	4,483 (38.6%)
	4	28,502 (24.0%)	4,774 (13.6%)	5,245 (18.9%)	8,703 (31.5%)	3,598 (21.5%)	6,182 (53.3%)
	Missing	2,188 (1.8%)	385 (1.1%)	400 (1.4%)	917 (3.3%)	259 (1.5%)	227 (2.0%)
**Sex**	Female	58,567 (49.4%)	18,156 (51.9%)	13,169 (47.5%)	13,232 (47.9%)	8,512 (50.8%)	5,498 (47.4%)
	Male	60,101 (50.6%)	16,860 (48.1%)	14,541 (52.5%)	14,368 (52.1%)	8,228 (49.2%)	6,104 (52.6%)
**In-hospital occupancy**	<95%	53,405 (45.0%)	16,266 (46.5%)	13,129 (47.4%)	12,880 (46.7%)	6,391 (38.2%)	4,739 (40.8%)
	95–100%	34,258 (28.9%)	10,452 (29.8%)	8,070 (29.1%)	7,930 (28.7%)	4,558 (27.2%)	3,248 (28.0%)
	100–105%	23,920 (20.2%)	6,569 (18.8%)	5,167 (18.6%)	5,251 (19.0%)	4,296 (25.7%)	2,637 (22.7%)
	>105%	7,085 (6.0%)	1,729 (4.9%)	1,344 (4.9%)	1,539 (5.6%)	1,495 (8.9%)	978 (8.4%)
**LWBS**	Yes	2,332 (2.0%)	692 (2.0%)	706 (2.5%)	571 (2.1%)	216 (1.3%)	147 (1.3%)
**Passed primary triage**	Yes	18,616 (15.7%)	4,103 (11.7%)	4,198 (15.1%)	5,778 (20.9%)	2,678 (16.0%)	1,859 (16.0%)
**Total visits**		118,668	35,016	27,710	27,600	16,740	11,602

Fractions rounded off to nearest decimal place.

Unadjusted analysis showed that the admitted fraction was smaller in strata of increasing in-hospital occupancy. For levels of in-hospital occupancy <95%, 95–100%, 100–105%, and >105% the admitted fractions were 31.5%, 30.9%, 29.9%, and 28.7%, respectively. The same pattern was observed in most of the subgroup analyses. Detailed results from the unadjusted analysis are reproduced in Table 2.

**Table 2:** Admitted fraction at different levels of in-hospital occupancy

		**<95%**	**95–100%**	**100–105%**	**>105%**	**Total**
**All units**	Admitted	16,845 (31.5%)	10,580 (30.9%)	7,159 (29.9%)	2,033 (28.7%)	36,617 (30.9%)
	Total	53,405	34,258	23,920	7,085	118,668
**Medicine unit**	Admitted	7,826 (48.1%)	4,831 (46.2%)	3,065 (46.7%)	801 (46.3%)	16,523 (47.2%)
	Total	16,266	10,452	6,569	1,729	35,016
**Surgery unit**	Admitted	4,358 (33.2%)	2,554 (31.6%)	1,718 (33.2%)	472 (35.1%)	9,102 (32.8%)
	Total	13,129	8,070	5,167	1,344	27,710
**Orthopaedics unit**	Admitted	1,959 (15.2%)	1,247 (15.7%)	694 (13.2%)	216 (14.0%)	4,116 (14.9%)
	Total	12,880	7,930	5,251	1,539	27,600
**Emergency physician unit**	Admitted	1,932 (30.2%)	1,424 (31.2%)	1,376 (32.0%)	428 (28.6%)	5,160 (30.8%)
	Total	6,391	4,558	4,296	1,495	16,740
**Otolaryngology**	Admitted	770 (16.2%)	524 (16.1%)	306 (11.6%)	116 (11.9%)	1,716 (14.8%)
	Total	4,739	3,248	2,637	978	11,602

All predictors screened for inclusion in the multivariate models were included because of clinical significance, except from time to physician, which was omitted due to violation of the assumption of linearity in the logit [31]. Table 3 shows the association between in-hospital occupancy level and probability for admission, with confounding factors from presenting complaint, age group, referral status, triage priority, presentation on a shift experiencing many visits, presentation on night shift/weekend, sex, LWBS, and entering the ED via primary triage taken into account.

**Table 3:** Odds ratios for admission, with confounding factors taken into account

	**Occupancy level**	**B**	**SE**	**Wald**	**Sig. ( *P *)**	**OR (CI)**
**All units**	<95% (ref)					1.00
	95–100%	−0.12	0.025	24.26	<0.001	0.88 (0.84–0.93)
	100–105%	−0.20	0.029	47.17	<0.001	0.82 (0.78–0.87)
	>105%	−0.31	0.046	43.96	<0.001	0.74 (0.67–0.81)
Nagelkerke R2	0.370					
**Medicine unit**	<95% (ref)					1.00
	95–100%	−0.14	0.030	22.60	<0.001	0.87 (0.82–0.92)
	100–105%	−0.22	0.036	36.46	<0.001	0.80 (0.75–0.86)
	>105%	−0.33	0.060	29.86	<0.001	0.72 (0.64–0.81)
Nagelkerke R2	0.367					
**Surgery unit**	<95% (ref)					1.00
	95–100%	−0.16	0.035	20.35	<0.001	0.85 (0.80–0.91)
	100–105%	−0.17	0.042	17.11	<0.001	0.84 (0.78–0.91)
	>105%	−0.17	0.070	6.10	0.014	0.84 (0.73–0.97)
Nagelkerke R2	0.261					
**Orthopaedics unit**	<95% (ref)					1.00
	95–100%	0.004	0.049	0.006	0.940	1.00 (0.91–1.11)
	100–105%	−0.26	0.061	18.62	<0.001	0.77 (0.68–0.87)
	>105%	−0.24	0.096	6.41	0.011	0.78 (0.65–0.95)
Nagelkerke R2	0.421					
**Emergency physician unit**	<95% (ref)					1.00
	95–100%	−0.006	0.049	0.016	0.899	0.99 (0.90–1.10)
	100–105%	−0.033	0.051	0.41	0.520	0.97 (0.88–1.07)
	>105%	−0.25	0.075	10.60	0.001	0.78 (0.68–0.91)
Nagelkerke R2	0.357					
**Otolaryngology unit**	<95% (ref)					1.00
	95–100%	0.002	0.12	0.000	0.989	1.00 (0.79–1.28)
	100–105%	−0.36	0.15	5.99	0.014	0.70 (0.53–0.93)
	>105%	−0.40	0.22	3.44	0.063	0.67 (0.44–1.02)
Nagelkerke R2	0.287					

Results from binary logistic regression models taking into account confounding from presenting complaint, age-group, referral status, triage priority, presentation on a shift experiencing many visits, presentation on night shift and during weekend, sex, leaving without being seen and entering ED via primary triage.

A clear association between increasing in-hospital occupancy and decreased odds ratio for admission was seen overall, with the odds ratios of admission being 0.88 (CI 0.84–0.93, *P* <0.001) for occupancy level 95–100%, 0.82 (CI 0.78–0.87, *P* <0.001) for occupancy level 100–105%, and 0.74 (CI 0.67–0.81, *P* <0.001) for occupancy level >105%, relative to the odds ratio for admission at occupancy level <95%. A similar pattern was observed upon subgroup analysis. Table 3 shows the coefficients of overall fit.

## Discussion

Both crude analysis and the adjusted analysis revealed a negative association between in-hospital occupancy and the odds ratio of patient admission. With few exceptions, the same pattern was observed upon subgroup analysis. The association between in-hospital occupancy level and decreased probability for admission established in the present study is supported by the findings of physicians aiming at reducing admissions when in-hospital beds are scarce [21,27].

Taking confounding from several factors into account, the multivariate models generated much information on factors associated with high probability of admission, which was omitted from this paper in order not to overshadow the main results. Most of the observed patterns were expected, i.e., increasing triage priority and age being associated with higher odds ratios of admission and different main complaints exhibiting different odds ratios for admission.

### Limitations

Despite its place on the study design hierarchy, the authors believe that this retrospective descriptive study was good at approximating reality as it included a large population with a wide range of complaints of varying severity, being treated by typical personnel. No bias is introduced through selection, which might be the case in controlled studies [32]. Considering the prevailing savings requirements at Helsingborg General Hospital, a rapid and inexpensive approach was also considered most ethical. The external validity of the results has to be met with a sound measure of scepticism, as the study was performed in a single hospital. Future studies should address this subject by comparing results between the different hospitals in the region.

Goodness-of-fit statistics indicate a limited ability of the multivariate models to predict admissions, suggesting that variables not included play a role. Previous studies indicate that co-morbidity is an important factor [33] and the authors believe that vital parameters would be desirable to include in future models. It would also be interesting to include effects of queuing for radiology or laboratory resources. However, the size and completeness of the data material should eliminate some of the bias from these factors and the authors wish to point out that the study objective was to reveal any correlation between in-hospital occupancy and the probability for admission, not to develop a tool for predicting admissions. For this purpose, the chosen method is adequate [34]. Age was divided into fairly few intervals in order to minimize risk for incomplete information from the variables. Given the large number of cases, this approach was too cautious and possibly concealed interesting findings in the population ≥65 years old.

## Conclusions

High in-hospital occupancy is associated with decreased odds ratios for admission of patients presenting in the ED of Helsingborg General Hospital during the study period. One interpretation is that a fraction of patients who would benefit from inpatient care instead received suboptimal care in outpatient settings at times of high in-hospital occupancy. The authors propose that downstream effects of such a relationship would be measurable, e.g., by increased incidence of unplanned revisits to the ED.

A second interpretation is that physicians admit patients who could be managed safely in the outpatient setting, in times of good in-hospital bed availability. Physicians thereby expose patients to healthcare-associated infections and other hazards, in addition to consuming resources better needed by others. A study addressing the first of these hypotheses is already initiated.

## Competing interests

The authors declare that they have no competing interests.

## Authors’ contributions

MB, KI and ML all participated in developing the study protocol. MB carried out gathering and matching of data and also carried out the statistical analyses. MB carried out the writing of the draft. All authors carried out proofreading of repeated versions of the manuscript.
